# Clinical and Radiological Significance of Anatomical Variations in Paranasal Sinuses: A Retrospective CT-Based Study

**DOI:** 10.7759/cureus.82506

**Published:** 2025-04-18

**Authors:** Nandha Kumar Subbiah, Satvinder S Bakshi, Sangeetha Arumugam, Joy A Ghoshal

**Affiliations:** 1 Department of Anatomy, All India Institute of Medical Sciences, Mangalagiri, Mangalagiri, IND; 2 Department of ENT, All India Institute of Medical Sciences, Mangalagiri, Mangalagiri, IND

**Keywords:** computed tomography (ct ), functional endoscopic sinus surgery (fess), paranasal air sinus, paranasal sinusitis, variations of anatomical structure

## Abstract

Background and objective

Anatomical variations of the paranasal sinuses influence sinus drainage and airflow, contributing to chronic rhinosinusitis. This study aimed to assess the prevalence of these variations and their association with sinus infections by using CT findings.

Methods

A retrospective CT-based analysis of 75 patients (aged 25-70 years) was conducted. Anatomical variations, including nasal septal deviation (NSD), concha bullosa, agger nasi cells, Haller cells, and Onodi cells, were recorded. The association between these variations and affected sinuses was analyzed using the chi-squared test and Fisher’s exact test; a p-value <0.05 was considered statistically significant.

Results

NSD was the most common anatomical variation, observed in 50 patients (66.6%), followed by hypertrophic ethmoidal bulla in 35 (46.6%), and concha bullosa in 30 (40%). Among the affected sinuses, maxillary sinusitis was the most frequently observed, affecting 60 patients (80%), followed by frontal in 45 (60%), ethmoidal in 30 (40%), and sphenoid sinusitis in eight (10.6%). Significant associations were found between NSD, concha bullosa, and maxillary sinusitis; agger nasi cells with frontal/ethmoid sinusitis; Haller cells with maxillary sinusitis; and Onodi cells with sphenoid sinusitis (p<0.05).

Conclusions

Paranasal anatomical variations significantly correlate with sinusitis, emphasizing the importance of preoperative CT evaluation in guiding medical and surgical management. Recognizing these variations enhances diagnostic accuracy and improves surgical outcomes in functional endoscopic sinus surgery (FESS).

## Introduction

The paranasal sinuses exhibit a wide range of anatomical variations that are crucial in clinical settings, particularly in the diagnosis and management of chronic rhinosinusitis and surgical planning for functional endoscopic sinus surgery (FESS) [1]. These variations are not only prevalent in the general population but also significantly influence sinonasal airflow, drainage patterns, and predisposition to pathological conditions [2]. Several studies have identified common anatomical variants, including nasal septal deviation (NSD), concha bullosa, Haller cells, Onodi cells, and variations in the uncinate process [3,4]. Among these, NSD is the most frequently observed, with reported prevalence rates ranging from 66.6% to over 80% in some populations [5]. NSD may contribute to sinus drainage obstruction and has been significantly associated with chronic maxillary sinusitis [6-8].

Concha bullosa, a condition characterized by pneumatization of the middle turbinate, is another common variation, observed in approximately 40-50% of cases and frequently linked to compromised airflow and sinus ostia obstruction [9]. Haller cells, located near the maxillary infundibulum, are implicated in maxillary sinusitis due to their potential to narrow the ostiomeatal complex, thereby impairing mucociliary clearance [10].

CT remains the gold standard imaging modality for identifying these anatomical variations. It provides high-resolution images that facilitate the evaluation of sinus drainage pathways, aiding in preoperative assessment and reducing intraoperative complications [11]. Studies have emphasized the importance of recognizing these variations to improve surgical outcomes and minimize risks such as orbital injury and cerebrospinal fluid leaks during sinus surgery [2,3,12]. This study aims to assess the prevalence and clinical implications of anatomical variations in the paranasal sinuses through a retrospective analysis of CT scans. We believe our findings will help establish associations between these variations and chronic rhinosinusitis, providing valuable insights for otolaryngologists and radiologists in both diagnostic and therapeutic contexts.

## Materials and methods

Study design and population

This hospital-based retrospective cross-sectional study was conducted in a tertiary care public hospital over five months in 2024-25 by the Department of Anatomy and the Department of ENT, All India Institute of Medical Sciences, Mangalagiri, India. Before the commencement of the study, ethical clearance was obtained from the Institutional Ethical Committee (AIIMS/MG/IEC/2024-25/83). CT images of the nose and paranasal sinuses, belonging to both male and female patients, were retrieved. A total of 75 CT scans of patients who were between 25-70 years were analyzed. 

Inclusion and exclusion criteria

Inclusion Criteria

Patients who underwent CT imaging of the paranasal sinuses for various clinical indications and presenting with symptoms of chronic rhinosinusitis or other sinonasal pathologies were included​ in the study.

Exclusion Criteria

Patients who had previously undergone surgery involving the nose or paranasal sinuses and individuals with paranasal sinus tumors, malignancies, or congenital anomalies affecting the nasal region were excluded. 

To minimize selection bias, all eligible patients who underwent CT imaging for non-traumatic, non-oncologic indications within the study period were included consecutively, thereby employing a convenience sampling strategy.

CT imaging protocol

CT scans were performed using a high-resolution multi-slice CT scanner following standardized imaging protocols. The axial images were acquired at 3 mm slice thickness in a cranio-caudal direction, covering the frontal sinus to the maxillary sinus, and later reconstructed into 0.6 mm multiplanar images. CT images were evaluated using coronal, axial, and sagittal planes, which enabled detailed analysis of sinonasal anatomical structures and variations. The radiological assessment was conducted using bone and soft tissue windows for better visualization of bony abnormalities and sinus opacification. 

Anatomical variations studied

The following anatomical variations were examined based on previous literature and their relevance to chronic rhinosinusitis and FESS: (1) Nasal septum: deviations (leftward, rightward, S-shaped), presence of bony spurs. (2) Turbinates: superior, middle, and inferior conchae, including pneumatization (concha bullosa) and paradoxical turbinates.​ (3) Paranasal sinuses: presence of hypoplasia or aplasia in the frontal, maxillary, sphenoid, and ethmoid sinuses. (4) Accessory air cells: presence of agger nasi, Onodi, and Haller cells, which are known to influence sinus drainage and airflow​

To reduce observer bias, all CT scans were independently reviewed by two specialists who were blinded to the clinical details of the patients. Discrepancies, if any, were resolved by consensus.

Statistical analysis

All findings were entered in a coded master sheet using Microsoft Excel, and statistical analysis was conducted using GraphPad Prism software. Categorical variables were summarized as frequencies and proportions. To determine the association between anatomical variations and affected paranasal sinuses, a chi-squared test was used to assess statistical significance, with Fisher’s exact test applied for smaller sample sizes where expected frequencies were low. A p-value <0.05 was considered statistically significant.

## Results

Demographic distribution

A total of 75 CT reports of patients diagnosed with rhinosinusitis were analyzed. The study population consisted of 45 males (60%) and 30 females (40%), with an age range of 25 to 75 years (mean age: 45 years).

Prevalence of anatomical variations

**Figure 1:**
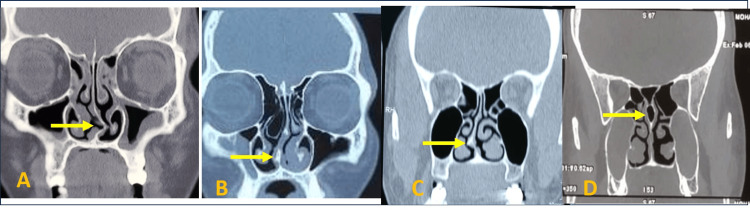
Coronal CT images of paranasal air sinuses showing nasal septal variations A: left-side deviation; B: S shape; C: spur; D: pneumatization CT: computed tomography

**Figure 2:**
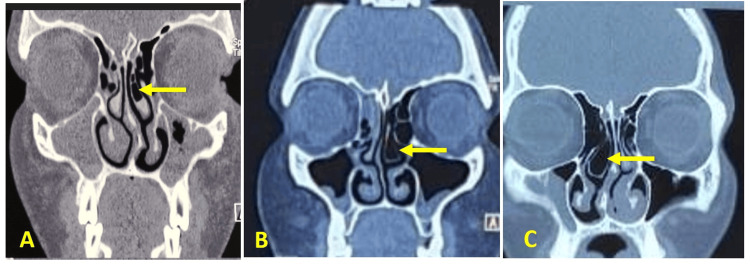
Coronal CT images of paranasal sinus showing variation in middle turbinate pneumatization A: lamellar type; B: bulbous type; C: extensive type (concha bullosa) CT: computed tomography

**Figure 3:**
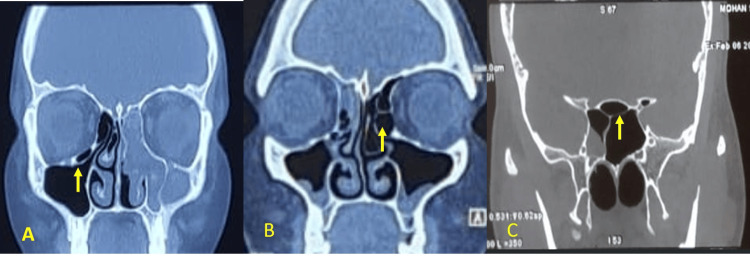
Coronal CT images of paranasal sinus showing types of air cells A: Haller cell; B: agger nasi cell; C: Onodi cell CT: computed tomography

**Figure 4:**
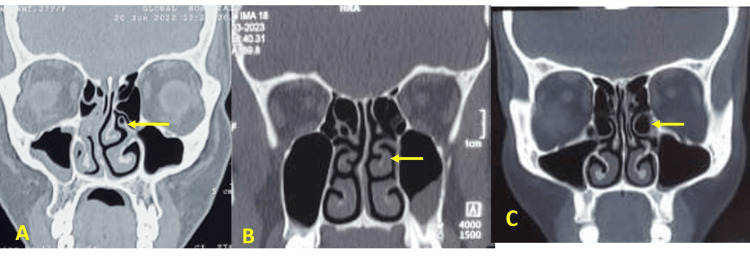
Coronal CT images of paranasal sinus showing other variations A: pneumatized uncinate process; B: bilateral paradoxical middle turbinate; C: left hypertrophied ethmoidal bulla CT: computed tomography

The CT scans revealed that anatomical variations were more commonly observed on the left side (66.6%) compared to the right side (33%). Bilateral variations were present in 35 cases (46.6%). The most frequently observed anatomical variation was NSD, present in 50 patients (66.6%) (Figure 1). Right-sided deviations were noted in 20 cases (26.7%), while left-sided deviations were seen in 30 cases (40%). Hypertrophic ethmoidal bulla was found in 46.6% (n=35) of cases, followed by concha bullosa of the middle turbinate in 40% (n=30) (Figure 2). Pneumatized nasal septum was noted in 20% (n=15), occurring unilaterally without bilateral involvement. Other variations included agger nasi cells (26.6%), Haller cells (20%), Onodi cells (13%) (Figure 3), paradoxical middle turbinate (10.6%) (Figure 4), frontal sinus hypoplasia (17%), maxillary sinus hypoplasia (6.6%), and sphenoid sinus hypoplasia (5%). Unilateral variations were more common than bilateral ones, with a higher prevalence on the left side (Table 1).

**Table 1:** Frequency distribution of affected paranasal sinuses

Common anatomic variations	Right side	Left side	Bilateral	Total (N=75)	Frequency (%)
Deviated Septum	20	30	0	50	66.6
Pneumatised Septum	15	15	0	15	20.0
Hypertrophic ethmoidal bulla	25	5	5	35	46.6
Middle turbinate (MT) Concha bullosa	15	10	5	30	40.0
Paradoxical MT	4	4	0	8	10.6
Pneumatised Uncinate Process	1	2	0	3	4.0
Frontal sinus Hypoplasia	4	6	3	13	17.0
Maxillary sinus Hypoplasia	3	2	0	5	6.6
Sphenoid sinus Hypoplasia	2	2	0	4	5.0
Agger Nasi Cell	5	10	5	20	26.6
Haller cell	7	5	3	15	20.0
Onodi Cell	5	3	2	10	13.0

Sinus involvement and clinical associations

The frequency distribution of paranasal sinuses affected in this study revealed that the maxillary sinus was the most commonly involved, affecting 60 cases (80%), followed by the frontal sinus in 45 cases (60%), anterior ethmoidal sinus in 30 cases (40%), posterior ethmoidal sinus in 10 cases (13%), and sphenoid sinus in eight cases (10.6%). Unilateral involvement was more frequent than bilateral, with right-sided involvement being predominant in the frontal (20 cases), anterior ethmoidal (15 cases), and posterior ethmoidal sinuses (five cases), whereas left-sided involvement was more common in the maxillary sinus (40 cases). Bilateral sinus involvement was observed in 10 cases of maxillary and frontal sinusitis and five cases of anterior ethmoidal sinusitis (Table 2).

**Table 2:** Distribution of common anatomical variations of the paranasal sinuses

Sinus affected	Right	Left	Total unilateral	Bilateral	Total, N (%)
Frontal	20	15	35	10	45 (60%)
Anterior ethmoidal	15	10	25	5	30 (40%)
Posterior ethmoidal	5	5	10	0	10 (13%)
Maxillary	10	40	50	10	60 (80%)
Sphenoid	5	3	8	0	8 (10.6%)

Association between anatomical variations and affected paranasal sinuses

The study identified significant associations between specific anatomical variations and affected paranasal sinuses by using the chi-squared test and Fisher’s exact test. Maxillary sinusitis was strongly associated with deviated septum (p<0.05), concha bullosa (p<0.05), and Haller cells (p<0.05), suggesting that structural abnormalities in nasal airflow and sinus drainage contribute to its pathogenesis. Frontal and ethmoid sinusitis were significantly associated with agger nasi cells (p<0.05), indicating their role in obstructing the frontal recess and affecting sinus ventilation. Onodi cells demonstrated a statistically significant association with sphenoid sinusitis (p<0.05), highlighting their clinical relevance due to their proximity to the optic nerve and sphenoid sinus (Table 3).

**Table 3:** Association between anatomical variations and affected paranasal sinuses

Presence of anatomical variation	Frontal sinus (n=45)	Ethmoid sinus (n=40)	Maxillary sinus (n=60)	Sphenoid sinus (n=8)	Chi-square	P-value
Deviated septum	13	9	39	6	14.34	<0.05
Concha bullosa	4	2	21	3	15.66	<0.05
Agger nasi cell	18	21	24	3	1.11	>0.05
Haller cell	6	1	12	1	5.5	>0.05
Onodi cell	1	9	3	7	43.6	<0.05

## Discussion

This study provides crucial insights into the prevalence and clinical significance of anatomical variations in the paranasal sinuses and their association with sinusitis. Potential confounding factors such as age, sex, and underlying sinonasal pathology were considered during data analysis to ensure accurate interpretation of anatomical variations. The most common anatomical variation observed was NSD (66.6%), aligning with prior studies reporting a prevalence range between 14.1% and 80% [5]. The significant association between deviated septum and maxillary sinusitis (p<0.05) observed in this study endorses findings in the literature that suggest that NSD obstructs normal sinus drainage and predisposes individuals to chronic rhinosinusitis [13]. Concha bullosa, detected in 40% of cases, is one of the most frequently reported variations in the literature, with previous studies citing a prevalence between 14% and 80% [9]. The significant correlation between concha bullosa and maxillary sinusitis (p<0.05) underscores its role in obstructing the middle meatus, leading to sinus drainage impairment, a phenomenon widely recognized in similar studies [13]. However, some researchers argue that concha bullosa may not always contribute to sinusitis unless significantly pneumatized [6,8,14,15].

Agger nasi cells, present in 26.6% of cases, showed a strong correlation with frontal sinusitis (p<0.05) and ethmoid sinusitis (p<0.05). This observation aligns with previous studies highlighting the role of agger nasi cells in frontal recess obstruction, contributing to chronic frontal sinus infections [16]. The importance of recognising agger nasi cells during FESS has been well documented due to their influence on the drainage pathway [17]. Haller cells, detected in 20% of cases, demonstrated a significant correlation with maxillary sinusitis (p<0.05). Haller cells have been implicated in narrowing the maxillary sinus infundibulum, leading to drainage obstruction, and our findings align with studies that establish their contribution to recurrent maxillary sinusitis [5,18]. Onodi cells, present in 13% of cases, were significantly associated with sphenoid sinusitis (p<0.05). Their clinical relevance is well established due to their close proximity to the optic nerve and internal carotid artery, making their recognition crucial in preventing surgical complications during FESS [13,19].

The associations established in this study emphasise the importance of preoperative imaging using CT scans in identifying anatomical variations that may predispose individuals to chronic sinus disease. The higher incidence of anatomical variations on the left side may be attributed to asymmetrical pneumatization patterns and developmental influences during craniofacial growth. The strong association between deviated nasal septum and maxillary sinusitis highlights the potential need for septoplasty in select patients suffering from recurrent maxillary sinus infections. Similarly, the presence of concha bullosa or Haller cells should raise suspicion for chronic maxillary sinus disease, and such patients may benefit from surgical interventions [9]. Furthermore, agger nasi cells should be carefully evaluated in patients with frontal sinusitis, as their presence often necessitates surgical correction to ensure optimal sinus drainage [16,18]. Onodi cells, due to their proximity to critical neurovascular structures, reinforce the necessity of careful preoperative assessment to avoid iatrogenic complications [20].

Limitations of the study 

Despite its valuable findings, this study has certain limitations. The sample size of 75 patients is relatively small compared to large-scale population-based studies. Larger, multicentric studies with diverse demographic groups would help validate these findings. The study is retrospective, limiting the ability to assess the functional impact of these variations beyond radiological analysis. Incorporating symptom correlation and endoscopic findings would enhance the understanding of the clinical relevance of these variations. Additionally, genetic and environmental factors influencing the prevalence of anatomical variations need further investigation.

## Conclusions

This study highlights the prevalence and clinical significance of anatomical variations in the paranasal sinuses and their association with chronic sinus disease. Significant correlations were established between deviated septum, concha bullosa, agger nasi cells, Haller cells, and Onodi cells with specific sinus pathologies, emphasizing their role in the pathogenesis of sinusitis. These findings underscore the importance of preoperative CT imaging in diagnosing sinonasal anatomical variations and guiding appropriate surgical interventions. Future research should focus on longitudinal studies, incorporating clinical symptom correlation, endoscopic evaluations, and larger sample sizes to further explore the functional implications of these variations. The integration of anatomical variation assessment in routine clinical practice can improve diagnostic accuracy, surgical outcomes, and overall patient care.
